# Calcium Signaling Regulated by Cellular Membrane Systems and Calcium Homeostasis Perturbed in Alzheimer’s Disease

**DOI:** 10.3389/fcell.2022.834962

**Published:** 2022-02-25

**Authors:** Dong-Xu Huang, Xin Yu, Wen-Jun Yu, Xin-Min Zhang, Chang Liu, Hong-Ping Liu, Yue Sun, Zi-Ping Jiang

**Affiliations:** ^1^ Department of Hand and Foot Surgery, The First Hospital of Jilin University, Changchun, China; ^2^ Department of Anesthesiology, The First Hospital of Jilin University, Changchun, China; ^3^ Department of Neurology, The First Hospital of Jilin University, Changchun, China; ^4^ Deparment of The First Operating Room, The First Hospital of Jilin University, Changchun, China

**Keywords:** calcium signaling, calcium homeostasis, endoplasmic reticulum, mitochondria, membrane contact site, Alzheimer’s disease

## Abstract

Although anything that changes spatiotemporally could be a signal, cells, particularly neurons, precisely manipulate calcium ion (Ca^2+^) to transmit information. Ca^2+^ homeostasis is indispensable for neuronal functions and survival. The cytosolic Ca^2+^ concentration ([Ca^2+^]_CYT_) is regulated by channels, pumps, and exchangers on cellular membrane systems. Under physiological conditions, both endoplasmic reticulum (ER) and mitochondria function as intracellular Ca^2+^ buffers. Furthermore, efficient and effective Ca^2+^ flux is observed at the ER-mitochondria membrane contact site (ERMCS), an intracellular membrane juxtaposition, where Ca^2+^ is released from the ER followed by mitochondrial Ca^2+^ uptake in sequence. Hence, the ER intraluminal Ca^2+^ concentration ([Ca^2+^]_ER_), the mitochondrial matrix Ca^2+^ concentration ([Ca^2+^]_MT_), and the [Ca^2+^]_CYT_ are related to each other. Ca^2+^ signaling dysregulation and Ca^2+^ dyshomeostasis are associated with Alzheimer’s disease (AD), an irreversible neurodegenerative disease. The present review summarizes the cellular and molecular mechanism underlying Ca^2+^ signaling regulation and Ca^2+^ homeostasis maintenance at ER and mitochondria levels, focusing on AD. Integrating the amyloid hypothesis and the calcium hypothesis of AD may further our understanding of pathogenesis in neurodegeneration, provide therapeutic targets for chronic neurodegenerative disease in the central nervous system.

## Introduction

The intraneuronal calcium ion (Ca^2+^) homeostasis is indispensable for neuronal functions and survival, even death (Miller, 1991; Berridge, 1998). Mainly, Ca^2+^ functions as a second messenger: the spatiotemporal change of the cytosolic Ca^2+^ concentration ([Ca^2+^]_CYT_), also known as the Ca^2+^ signal, is one of the ways that cells convey various information either intracellularly or intercellularly (Berridge et al., 1998). Additionally, Ca^2+^ acts as a carrier of positive electrical current, which enters into the cytosol and depolarizes the transmembrane potential (Byrne et al., 2014).

**FIGURE 1:**
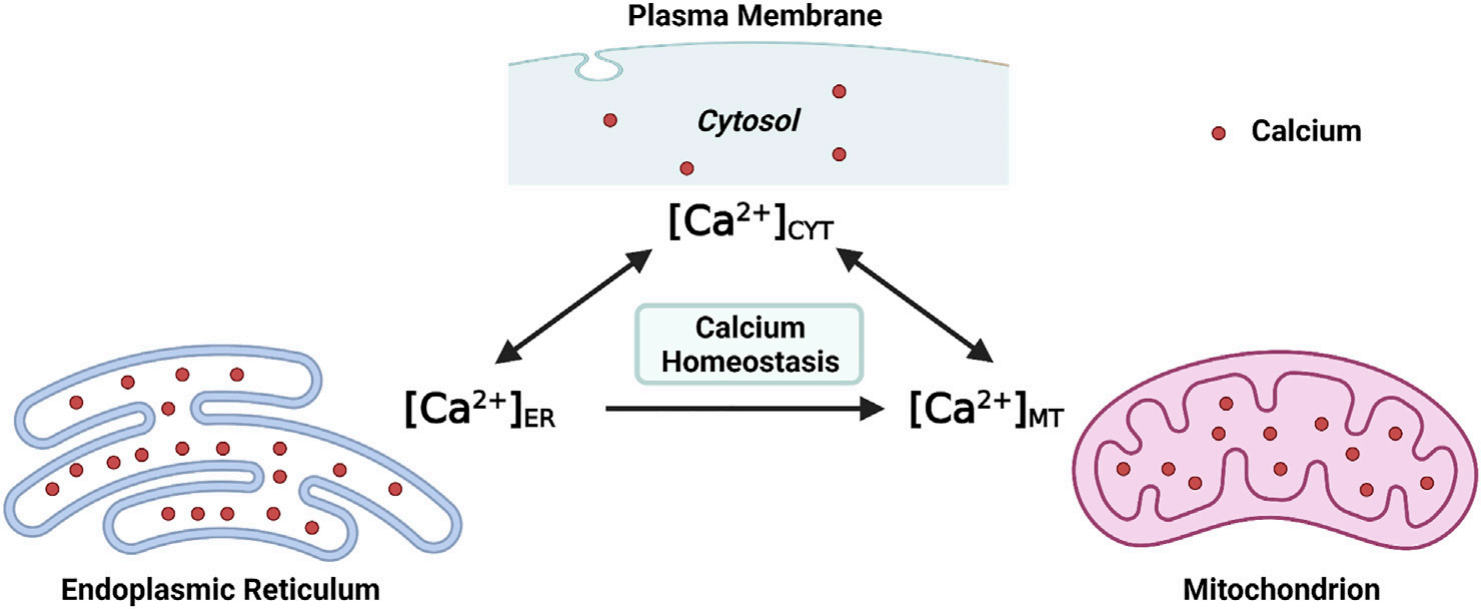
Maintaining the intraneuronal calcium homeostasis: the endoplasmic reticulum intraluminal Ca^2+^ concentration ([Ca^2+^]_ER_) and the mitochondrial matrix Ca^2+^ concentration ([Ca^2+^]_MT_) fluctuate with the cytosolic Ca^2+^ concentration ([Ca^2+^]_CYT_) (Created with BioRender.com).

At the molecular level, the [Ca^2+^]_CYT_ is regulated by channels, ATPase pumps, and ion exchangers on cellular membrane systems (the plasma membrane and intracellular membranes), as well as Ca^2+^-binding proteins in the cytosol (Byrne et al., 2014). At the subcellular level, at least two organelles, endoplasmic reticulum (ER) and mitochondria, have participated in the regulation of [Ca^2+^]_CYT_ either respectively or interactively (Martonosi, 1984; Miller, 1991; Spät et al., 2008). Structurally, the ER extends into every inner domain in neurons, and mitochondria tend to localize in intraneuronal compartments that consume massive ATPs, such as synapses (Sheng and Cai, 2012; Wu et al., 2017). Functionally, both the ER and mitochondria act as internal Ca^2+^ sources and sinks; namely, both organelles possess the role of buffering the [Ca^2+^]_CYT_ (Miller, 1991; Berridge, 1998; Spät et al., 2008). Collectively, both the endoplasmic reticulum intraluminal Ca^2+^ concentration ([Ca^2+^]_ER_) and the mitochondrial matrix Ca^2+^ concentration ([Ca^2+^]_MT_) fluctuate simultaneously with [Ca^2+^]_CYT_ (Figure 1). Moreover, efficient and effective Ca^2+^ flux is observed at the ER-mitochondria contact site (ERMCS), where the two organelles are intimately apposed (Wu et al., 2018). Briefly, Ca^2+^ is released from the ER lumen followed by mitochondrial Ca^2+^ uptake into the mitochondrial matrix through the outer and inner mitochondrial membranes in sequence (Rizzuto et al., 2012).

Maintaining the physiological level of [Ca^2+^]_CYT_, [Ca^2+^]_ER_, and [Ca^2+^]_MT_ is essential for intraneuronal Ca^2+^ homeostasis. When the neuronal Ca^2+^ signaling is dysregulated, neurons will undergo excitotoxicity or apoptosis (Lipton and Rosenberg, 1994; Berridge et al., 1999). The intraneuronal Ca^2+^ dyshomeostasis contributes to neurodegenerative diseases such as Alzheimer’s disease (AD), an irreversible chronic neurodegenerative disease without effective treatment (Pchitskaya et al., 2018). The underlying cellular and molecular mechanisms which regulate Ca^2+^ signaling and maintain intracellular Ca^2+^ homeostasis, particularly by the ER and mitochondria, are summarized in the present review, focusing on AD.

## Endoplasmic Reticulam is the Calcium Source Inside the Neuron

### Subcellular Structures Formed by ER in the Neuron

The ER extends into every portion of the neuron to form an elaborate network, also considered as “a neuron within a neuron” (Berridge, 1998; Wu et al., 2017). The ER membrane, which connects with the nuclear envelope, also connects with the plasma membrane to form various types of specialized regions named the subsurface cisternae (located in the soma and initial dendrites, similar to the triadic junction in myocytes), the cisternae organelle (multilayered subsurface cisternae situated in the initial segment of the axon), the hypolemmal cisternae (located in the axon), and the spine apparatus (located in the dendritic spine) (Berridge, 1998).

### Two Primary ER Ca^2+^ Channels: InsP_3_R and RyR

#### Types and Distribution of ER Ca^2+^ Channels

As in other cell types, neuronal ER also contains the inositol 1,4,5-triphosphate receptor (InsP_3_R) and the ryanodine receptor (RyR), sharing similar characteristics (Galione et al., 1993; Striggow and Ehrlich, 1996). Structurally, InsP_3_Rs are homo- or hetero-tetrameric assemblies that own three isoforms, namely type 1 (InsP_3_R1), type 2 (InsP_3_R2), type 3 (InsP_3_R3) (Taylor, 1998; Spät et al., 2008). Similarly, RyRs are tetrameric proteins that possess three subtypes: RyR1, the skeletal muscle type; RyR2, the cardiac muscle type; RyR3, the brain type (Querfurth et al., 1997; Spät et al., 2008). Functionally, InsP_3_Rs and RyRs are chemically-gated Ca^2+^ channels that evoke the regenerative Ca^2+^ wave from the ER lumen to the cytosol, also known as the Ca^2+^-induced Ca^2+^ release (CICR) (Martonosi, 1984; Berridge, 1998; Spät et al., 2008). Seemingly, InsP_3_Rs and RyRs have evolved from the same ancestor owing to the similarities (Berridge, 1997).

Spatially, InsP_3_Rs and RyRs share similar but not identical distributions in neurons (Berridge, 1998). From the subcellular perspective, InsP_3_Rs spread widely within the neuron, while RyRs localize predominantly in the soma (Walton et al., 1991; Kuwajima et al., 1992; Takei et al., 1992). Concerning mouse hippocampal neurons, both RyRs and InsP_3_Rs coexist densely within the soma; but are distributed heterogeneously within dendrites: RyRs are restricted to the proximal region of dendrites, InsP_3_Rs are found in the whole region of dendrites (Seymour-Laurent and Barish, 1995). Intriguingly, inspecting dendrites of chicken cerebellum Purkinje cells, there are only InsP_3_Rs and no RyRs within the dendritic spine, but there are both InsP_3_Rs and RyRs within the dendritic shaft (Walton et al., 1991). From the anatomical perspective, the cardiac muscle type RyR2, which conducts the Ca^2+^-elicitated Ca^2+^ release, is detected throughout the brain; nevertheless, the skeletal muscle type RyR1, which performs the depolarization-evoked Ca^2+^ release, is seen exclusively in the cerebellum; the brain type RyR3 is distributed within the hippocampus, cortex, and corpus striatum (Kuwajima et al., 1992; Querfurth et al., 1997).

#### Elementary and Global Ca^2+^ Signals from ER

Neuronal Ca^2+^ signal initiates with increasing of [Ca^2+^]_CYT_, which is followed by decreasing of [Ca^2+^]_CYT_ to the resting level (Miller, 1991). Although various types of Ca^2+^ signals are named in different ways, it is less important to focus on the terminology but essential for identifying their characteristics (Berridge et al., 1999).

The elementary Ca^2+^ signals originating from ER Ca^2+^ channel own hierarchical characteristics (Bootman et al., 1997; Berridge et al., 1999). At the fundamental level, the “blip” from InsP_3_R and the “quark” from RyR are analogous, both of which are evoked from a single channel (Bootman et al., 1997). At the intermediate level, the “puff” from InsP_3_Rs and the “spark” from RyRs are similar, both of which are liberated from clusters of channels (Bootman et al., 1997). These elementary Ca^2+^ signals are characterized by a quick rise period followed by a slow recovery period (Berridge, 1997). The underlying mechanism is that the opening of the channel leads to a plume of Ca^2+^ releasing from ER lumen; after the channel’s closing, the released Ca^2+^ plume dissipates slowly by diffusion (Berridge, 1997).

These elementary Ca^2+^ signals construct the global Ca^2+^ signals, such as waves (at the subcellular level) and oscillations or spikes (at the whole-cell level) (Bootman et al., 1997; Berridge et al., 1999). Ca^2+^ waves propagate by regional Ca^2+^ diffusions and neighbor Ca^2+^ regenerations, based on the CICR, a positive feedback mechanism (Bootman et al., 1997). Furthermore, CICR is regulated by the positive and negative feedback influence of Ca^2+^ on the InsP_3_R or RyRs, which are discussed later (Berridge, 1997). Under high, intermediate, low positive feedback CICR, the Ca^2+^ waves, respectively, are continuous, saltatory, and abortive (Bootman et al., 1997).

#### Regulation of InsP_3_R Ca^2+^ Channel

The Ca^2+^-release activity from the opened InsP_3_R, at least, is regulated by the InsP_3_, [Ca^2+^]_CYT_, and [Ca^2+^]_ER_. Under a modest concentration of InsP_3_, the opening of InsP_3_R is biphasically regulated by cytosolic Ca^2+^: the low [Ca^2+^]_CYT_ (<1 μM) can activate InsP_3_R; in contrast, the high [Ca^2+^]_CYT_ (>1–10 μM) can inhibit the channel (Bootman and Lipp, 1999). Under the circumstance mentioned above, the original graph describing the probability of the InsP_3_R opening against the [Ca^2+^]_CYT_ level reveals a bell-shaped curve (Bootman and Lipp, 1999). The ascending portion of the bell-shaped curve yields the positive feedback effect of the [Ca^2+^]_CYT_ on the InsP_3_R opening, which allows the localized elementary Ca^2+^ signal to spread regeneratively as Ca^2+^ waves (Berridge, 1997; Sun et al., 1998). The descending portion of the bell-shaped curve represents the negative feedback dependence of the InsP_3_R opening on the [Ca^2+^]_CYT_, which terminates the elementary Ca^2+^ signal (Berridge, 1997; Sun et al., 1998).

Constructively, Adkins and Taylor suggest that InsP_3_ acts as a molecular switch that converts the InsP_3_R from a condition under which only an inhibitory Ca^2+^-binding site is feasible to one under which only a stimulatory Ca^2+^-binding site is viable (Adkins and Taylor, 1999). Sequentially, two steps are required for opening the InsP_3_R: initially, it becomes a liganded InsP_3_R by binding with InsP_3_; subsequently, it becomes an active InsP_3_R by binding with Ca^2+^ at the stimulatory Ca^2+^-binding site (Adkins and Taylor, 1999).

Nevertheless, the bell-shaped dependence of the InsP_3_R opening on the [Ca^2+^]_CYT_ is not always expected. If the high [Ca^2+^]_CYT_ (100 μM) is applied secondary to the maximal concentration of InsP_3_ (10 μM), the cytosolic Ca^2+^ fails to inhibit the Ca^2+^ release from the liganded InsP_3_R; in turn, if the high [Ca^2+^]_CYT_ (100 μM) is given before the InsP_3_ (10 μM), the cytosolic Ca^2+^ can entirely inhibit the Ca^2+^ release from the unliganded InsP_3_R (Adkins and Taylor, 1999). Moreover, the liganded InsP_3_R owns a limited time window beyond which it undergoes intrinsic inactivation, and then the cytosolic Ca^2+^ cannot activate the InsP_3_R (Bootman and Lipp, 1999). Notably, although the opening of InsP_3_R requires binding with both InsP_3_ and Ca^2+^, it might not necessarily need the cytosolic Ca^2+^ (Bootman and Lipp, 1999). When [Ca^2+^]_ER_ is low, the opening of InsP_3_R requires both InsP_3_ and cytosolic Ca^2+^; however, when [Ca^2+^]_ER_ is high, there is no requirement for cytosolic Ca^2+^, it is enough for InsP_3_ itself to open the InsP_3_R (Missiaen et al., 1994).

Collectively, at the high InsP_3_ level and the low [Ca^2+^]_ER_ level, the high [Ca^2+^]_CYT_ cannot inhibit InsP_3_R because most InsP_3_Rs are liganded (Adkins and Taylor, 1999). At the low InsP_3_ level and the high [Ca^2+^]_ER_ level, the low [Ca^2+^]_CYT_ cannot activate InsP_3_R due to InsP_3_ alone can open the InsP_3_R (Missiaen et al., 1994).

#### Regulation of RyR Ca^2+^ Channel

The RyR is opened and releases Ca^2+^ into the cytosol by Ca^2+^ binding with the high-affinity stimulatory site; the Ca^2+^ is released until the local [Ca^2+^]_CYT_ rises to the point where the low-affinity inhibitory site is bound, resulting in the RyR closing, which is the mechanism of CICR mediated by RyR (Payne et al., 2013). RyR1 and RyR2 are studied extensively in skeletal myocyte and cardiac myocyte, respectively. Dihydropyridine receptor (DHPR)-coupled RyR1 is opened upon depolarization of the plasma membrane and then is closed upon repolarization; subsequently, the surrounding uncoupled RyR1 is regeneratively opened under the CICR mechanism (Berridge, 1997). RyR2 is opened by the brief cytosolic Ca^2+^ pulse from DHPR, which is activated upon depolarization of the plasma membrane; approximately four RyR2s together evoke the Ca^2+^ quark, then these quarks turn to sparks, finally to waves (Berridge, 1997). Additionally, the activation of RyR is also regulated by the [Ca^2+^]_ER_ level (Györke and Györke, 1998). Similar to InsP_3_R, when [Ca^2+^]_ER_ is overloaded, the Ca^2+^-release activity of RyR is also significantly potentiated (Cheng et al., 1996).

## Mitochondria are Calcium Buffers Inside the Neuron

### Mitochondria-Linked Cytosolic Ca^2+^ Buffering

In addition to synthesizing adenosine triphosphate (ATP), another primary function of mitochondria is buffering intracellular Ca^2+^ (Miller, 1991). Neuronal mitochondria segregate Ca^2+^ under both physiological and pathological conditions (Miller, 1991). The Ca^2+^ buffering ability of mitochondria may lead to the accumulation of abundant Ca^2+^ in a particular domain in neurons (Rizzuto et al., 2012). Mitochondria may function as the last line against the exaggerated [Ca^2+^]_CYT_, which may be fatal for cells when other intracellular Ca^2+^-regulating mechanisms are exhausted (Martonosi, 1984). It is considered that the majority of mitochondria are generated in the soma, and the dysfunctional mitochondria return to the soma for degradation (Sheng and Cai, 2012).

Mitochondria usually cluster in neuronal domains with high demand for ATP, such as presynaptic and postsynaptic terminals (Tang and Zucker, 1997). In neurons, mitochondria located in proximal to Ca^2+^ channels, such as NMDAR on the postsynaptic density, can accumulate the cytosolic Ca^2+^ and prevent the propagation of Ca^2+^ waves, a global Ca^2+^ signal (Rizzuto et al., 2012). In the post-tetanic potentiation, mitochondria in the presynaptic terminal regulate the [Ca^2+^]_CYT_ by buffering extra intraneuronal Ca^2+^: during tetanic stimulation, mitochondria take up Ca^2+^; after tetanic stimulation, mitochondria release Ca^2+^ into the cytosol, maintaining the [Ca^2+^]_CYT_ at a relatively high level (Tang and Zucker, 1997).

### Mitochondria-Located Ca^2+^ Machinery

Logically, the entrance of Ca^2+^ into the mitochondrial matrix requires passing through two intracellular membranes: the outer mitochondrial membrane (OMM) and the inner mitochondrial membrane (IMM). The OMM is permeable to ions attributed to the massive expression of voltage-dependent anion channels (VDAC) (Rizzuto et al., 2012). The notion that the expression level of VDACs seems to be the bottleneck of mitochondrial Ca^2+^ uptake is supported by the demonstration that over-expression of VDACs potentiates [Ca^2+^]_MT_; in contrast, down-regulation of VDACs attenuates [Ca^2+^]_MT_ (Madesh and Hajnóczky, 2001; Rapizzi et al., 2002). Among three isoforms of VDACs (VDAC1, VDAC2, VDAC3), the VDAC1 isoform selectively interacted with InsP_3_R3 to transmit Ca^2+^ signal into the mitochondrial matrix that associates with apoptosis (De Stefani et al., 2012). Consistently, in the Chinese hamster ovary cell models that express all three isoforms of InsP_3_Rs, the InsP_3_R3 preferentially conducts Ca^2+^ signal into the mitochondria to induce apoptosis (Mendes et al., 2005).

The mitochondrial calcium uniporter (MCU) on the IMM can rapidly accumulate Ca^2+^ into the mitochondria matrix across the electrochemical gradient (Gunter and Gunter, 1994). MCU selectively binds Ca^2+^ with extremely high affinity (K_D_ ≤ 2 nM) (Kirichok et al., 2004). MCU contains two transmembrane domains and significantly potentiates mitochondrial Ca^2+^ uptake after over-expression (De Stefani et al., 2011). Acidic residues, a binding site for ruthenium red and its analogs (the most potent inhibitors of MCU), reside in the highly conserved motif between the two transmembrane domains and are essential for the entire activity of MCU (Baughman et al., 2011). The mitochondrial calcium uptake 1 (MICU1) protein interacts directly with MCU to regulate the rapid Ca^2+^ uptake of mitochondria (Perocchi et al., 2010).

## Calcium Cross-Talk Through Endoplasmic Reticulam-Mitochondria Contact Site

The ER has distributed the entire intracellular space from the nucleus to the plasma membrane intertwining all organelles, including mitochondria (Giorgi et al., 2009; Lebiedzinska et al., 2009; Wu et al., 2018). The ER network in which mitochondria are embedded exists in all compartments of neurons (Wu et al., 2017; Wu et al., 2018). The endoplasmic reticulum-mitochondria contact site (ERMCS) is abundant in every neuronal domain from the soma to dendrites and the axon (Wu et al., 2017). The distance between the two membranes in ERMCS is less than 200 nm (Rizzuto et al., 1998). Since the early 1960s, several different contact sites between the opposing membranes have been identified, such as the plasma membrane-ER contact site and the plasma membrane-mitochondria contact site (Lebiedzinska et al., 2009).

**FIGURE 2:**
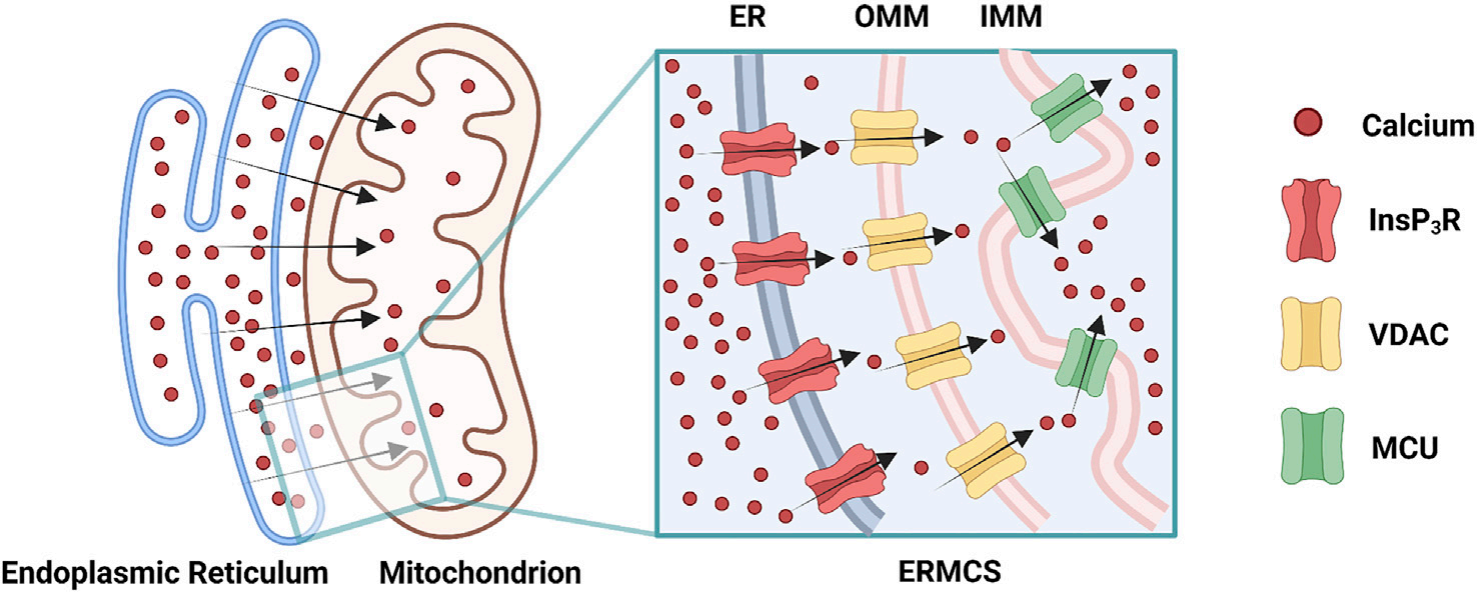
Ca^2+^ transmits through the endoplasmic reticulam-mitochondria contact site (ERMCS): Ca^2+^ releases from the endoplasmic reticulam (ER) membrane via the inositol 1,4,5-triphosphate receptor (InsP_3_R), followed by mitochondrial Ca^2+^ uptake by the voltage-dependent anion channel (VDAC) on the outer mitochondrial membrane (OMM), subsequently by the mitochondrial calcium uniporter (MCU) on the inner mitochondrial membrane (IMM) (Created with BioRender.com).

Mitochondrial Ca^2+^ uptake can occur at the ERMCS, where a high concentration of Ca^2+^ transports from the ER lumen into the mitochondrial matrix (Rizzuto et al., 2012). Briefly, Ca^2+^ releases from ER membrane via the InsP_3_R, followed by mitochondrial Ca^2+^ uptake by the VDAC on OMM, subsequently by the MCU on the IMM (Figure 2) (Rizzuto et al., 2012). Mitochondrial Ca^2+^ uptake can regulate the activity of InsP_3_R by decreasing the [Ca^2+^]_CYT_ nearby ER membrane (Rizzuto et al., 2012). During Ca^2+^ absorbing by mitochondria, the [Ca^2+^]_CYT_ near the InsP_3_R mouth is not high enough to block the channel; hence the InsP_3_R sustain opening, and the Ca^2+^ release from ER is prolonged (Boitier et al., 1999; Hajnóczky et al., 1999; Rizzuto et al., 2012). The increased ERMCS may induce the mitochondrial Ca^2+^ overload following Ca^2+^ release from the ER; conversely, the decreased ERMCS may impair Ca^2+^-dependent mitochondrial metabolism (Csordás et al., 2006; Lebiedzinska et al., 2009). As mentioned before, InsP_3_R3-VDAC1 interaction seems to play a major role in Ca^2+^ fluxion in ERMCS (Mendes et al., 2005; De Stefani et al., 2012). Collectively, [Ca^2+^]_ER_, [Ca^2+^]_CYT_, and [Ca^2+^]_MT_ are simultaneously regulated by ERMCS.

## Alzheimer’s Disease: Irreversible Neurodegeneration Without Effective Therapies

### Characteristics of Alzheimer’s Disease

Alzheimer’s disease (AD), first described in 1907 (Alzheimer et al., 1995), is a type of chronic neurodegenerative disease growing in number, which has brought physical sufferings, psychological stresses, and economic burden to individuals, families, and society (Alzheimer’sAssociation, 2020). Regrettably, there are no available medications for slowing, ceasing, or reversing the neuronal pathological progression that causes neurodegenerative symptoms and makes AD fatal (Alzheimer’sAssociation, 2020). Merely five drugs improving symptoms of AD have been approved by the Food and Drug Administration (FDA): three cholinesterase inhibitors (galantamine, rivastigmine, donepezil); one NMDAR blocker (memantine); one concomitant agent (memantine and donepezil) (Kumar et al., 2015; Atri, 2019; Alzheimer’sAssociation, 2020). Additionally, tacrine, a cholinesterase inhibitor approved once by FDA, is discontinued in the United States due to severe side effects, such as liver damage (Kumar et al., 2015; Alzheimer’sAssociation, 2016). Until 2021, 126 agents are in clinical trials for AD in the United States, and most investigational new drugs target modification of AD (Cummings et al., 2021). Recently, the repurposing and repositioning of conventional drugs is considered an alternative strategy for cancer therapy (Heckman-Stoddard et al., 2017; Huang et al., 2021). The same strategy could facilitate the identification of novel therapy for AD (Ballard et al., 2020).

Pathologically, the senile plaques (also known as β-amyloid plaques or neuritic plaques) and the neurofibrillary tangles (NFT) (also known as tau tangles or dystrophic neurites), observed inside and outside neurons, respectively, are two of several neuropathological features related to AD (Selkoe and Hardy, 2016; Alzheimer’sAssociation, 2020).

Based on the age of morbidity, Alzheimer’s disease is divided into two subtypes: the early-onset AD (EOAD), ranging from 30 years to 60 or 65 years; the late-onset AD (LOAD), defined with an onset age later than 60 or 65 years (Bekris et al., 2010). At the inheritance level, EOAD is characterized by the hereditary form, also known as the familial AD (FAD); by contrast, LOAD is typically termed as the sporadic AD (SAD) (Selkoe and Hardy, 2016; Kozlov et al., 2017).

### Genetics of Alzheimer’s Disease

Mutations in the *amyloid precursor protein* (*APP*), *presenilin-1* (*PSEN1*), *and presenilin-2* (*PSEN2*) genes are genetically associated with FAD (Bekris et al., 2010). The *APP* gene resides on chromosome 21 (Selkoe, 1994). Indeed, individuals with Down syndrome (DS) have an increased risk of developing AD owing to trisomy 21 (Alzheimer’sAssociation, 2020). The *PSEN1* gene, residing in chromosome 14, encodes the presenilin-1 protein of 467 amino acids which contains nine transmembrane domains; the *PSEN2* gene, residing in chromosome 1, encodes the presenilin-2 protein of 448 amino acids topologically 67% identical to the presenilin-1 protein (Levy-Lahad et al., 1995; Sherrington et al., 1995; Cook et al., 1996; Leissring et al., 1999a; Laudon et al., 2005; Bekris et al., 2010). Mutations in the *APP* gene account for less than 5% of all FAD cases, mutations in the *PSEN1* gene are responsible for approximately 70% of early-onset FAD (Van Broeckhoven, 1995). Consequently, mutations in the *PSEN1* gene are the most common cause of presenile FAD; by contrast, mutations in the *PSEN2* gene are a rare cause (Bekris et al., 2010). Mutations in the *apolipoprotein E* (*APOE*) gene, residing in chromosome 19, fulfill a significant role in SAD (Bertram and Tanzi, 2004; Bekris et al., 2010). Less than one hundred families with mutations in the *APP* gene, as well as several hundred families with mutations in the *PSEN1* gene and the *PSEN2* gene have been reported worldwide, hence the FAD cases would occur in less than 1% of all AD cases (Bekris et al., 2010; Castellani and Smith, 2011). More than 90% of individuals with AD would suffer the sporadic type of this disease (Bekris et al., 2010).

## Integrating Amyloid Hypothesis and Calcium Hypothesis of Alzheimer's Disease

Following the “amyloid hypothesis” of AD, initiated by the study of Glenner and Wong in 1984, the accumulation of the amyloid-β (Aβ) peptide is the predominant force of AD-related pathogenesis, including plaques, tangles, synapse loss, and neuronal death (Glenner and Wong, 1984; Tanzi and Bertram, 2005). Although there are still several controversies (Castellani and Smith, 2011; Kozlov et al., 2017), the amyloid hypothesis, supported by many preclinical and clinical studies, has become the primary model of AD pathogenesis and has provided potential therapeutic targets for AD treatments (Selkoe and Hardy, 2016).

The “calcium hypothesis” of AD, which regards the persistent intraneuronal Ca^2+^ dyshomeostasis as one of the early causes of AD, is first proposed by Khachaturian based on limited direct evidence in the 1980s (Khachaturian, 1994; LaFerla, 2002). Growing lines of evidence have emerged to support the calcium hypothesis (Mattson et al., 2000). Ca^2+^ regulates a series of neuronal functions, such as neurotransmitter release and synaptic plasticity; in turn, neurons own precise mechanisms to sustain the Ca^2+^ homeostasis (LaFerla, 2002). For the intraneuronal Ca^2+^ dyshomeostasis to trigger the AD pathology, the Ca^2+^ signal perturbation must be an initial phenotype of AD, and the Ca^2+^ signaling dysregulation can affect the Aβ accumulation and the tau protein hyperphosphorylation (LaFerla, 2002). Although the former is still controversial (LaFerla, 2002), the latter is well accepted by viable evidence (Mattson, 1990; Mattson et al., 1993).

The relationship between the amyloid hypothesis and other potential hypotheses of AD may not conflict with one theory against another (Selkoe and Hardy, 2016). Moreover, integrating the amyloid hypothesis (Hardy and Selkoe, 2002; Bekris et al., 2010) and the calcium hypothesis (LaFerla, 2002) may further the understanding of Alzheimer’s disease pathogenesis. The calcium hypothesis remains compelling, and targeting selective calcium pathways would be a competitive therapeutic approach for AD (LaFerla, 2002).

## Amyloid-B Peptide is Associated With Calcium Dyshomeostasis in Alzheimer's Disease

### Aβ Forms Ca^2+^-Permeable Channel

**FIGURE 3:**
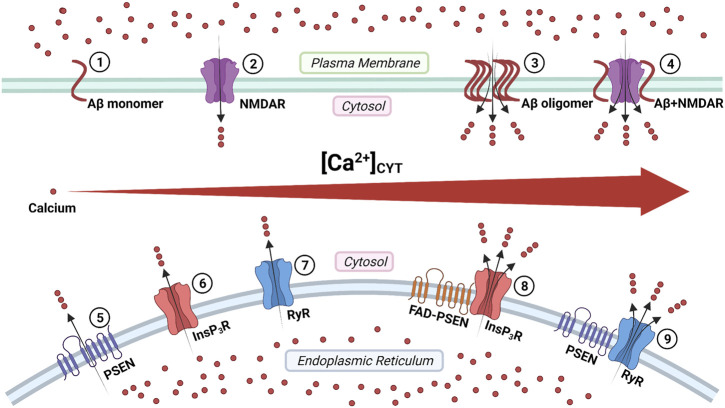
The regulation of calcium signaling by cellular membrane systems: ①③ amyloid-β (Aβ) peptide monomers aggregate into the oligomer which forms Ca^2+^-permeable channel; ②④ the Aβ accumulation promotes the persistent Ca^2+^ signal through the N-methyl-_D_-aspartate receptor (NMDAR); ⑤ the presenilin (PSEN) holoprotein functions as the endoplasmic reticulum (ER) passive Ca^2+^ leak channel; ⑥⑧ the enhanced Ca^2+^ signal by the familial Alzheimer's disease- causing mutant presenilin (FAD-PSEN) is the inositol 1,4,5-triphosphate receptor (InsP_3_R) dependent; ⑦⑨ the interaction between the PSEN and the ryanodine receptor (RyR) regulates the Ca^2+^ signal (Created with BioRender.com).

Aβ peptides form Ca^2+^-permeable channels (also known as Aβ channels) on the plasma membrane and disrupt Ca^2+^ homeostasis by rapidly elevating intracellular Ca^2+^ concentration, leading to neuronal death in AD (Figure 3) (Arispe et al., 1993; Arispe et al., 1994b). The physical and chemical characteristics of Aβ peptides enable the formation of the β-sheet and subsequent aggregation into dimers and, even, large oligomers, which form β-barrel structures for the cation-selective permeability, particularly for Ca^2+^ (Figure 3) (Kagan et al., 2002). The nanomole (nM)-level concentrations of Aβ_42_ can form Ca^2+^-permeable channels, which elevate [Ca^2+^]_CYT_ levels and rapidly elicit the degeneration of cultured endothelial cells (Bhatia et al., 2000). When incorporating Aβ_40_ into the artificial bilayer membrane, Ca^2+^ permeates through the opened Aβ channels (Arispe et al., 1993). The Ca^2+^ influxes through these channels would prevail due to the most significant electrochemical gradient between extracellular Ca^2+^ concentration and [Ca^2+^]_CYT_ (Arispe et al., 1993; Arispe et al., 1994a). For a neuron with a single Aβ channel in opening state, the corresponding Ca^2+^ influx would increase the [Ca^2+^]_CYT_ level at a rate of 5 μmol per second (5 μM/s), exhausting the neuronal Ca^2+^ buffering capacity rapidly, subsequently leading to the neurotoxicity (Arispe et al., 1993; Arispe et al., 1994a).

### Aβ Activates NMDAR

The N-methyl-_D_-aspartate receptor (NMDAR) is named by its specific agonist, N-methyl-_D_-aspartate (NMDA), which does not occur naturally. NMDARs belong to one ionotropic family of glutamate receptors located on the plasma membrane. NMDARs can integrate two extracellular chemical stimuli (glycine and glutamate) and one membrane electrical stimulus (the depolarization of the plasma membrane) into the Ca^2+^ signal (Lipton and Rosenberg, 1994; Furukawa et al., 2005). Structurally, NMDARs constitute three families of subunits: glycine-binding NR1, which owns eight isoforms; glutamate-binding NR2, including NR2A, NR2B, NR2C, and NR2D; glycine-binding NR3, including NR3A and NR3B (Cull-Candy and Leszkiewicz, 2004; Furukawa et al., 2005). Functional NMDARs are tetrameric assemblies composed of two copies of NR1/NR2 heterodimers, sometimes NR1/NR3 heterodimers (Chen and Wyllie, 2006). Moreover, identical or diverse NR2 subunits form di-heteromeric assemblies (such as NR1-NR1-NR2A-NR2A, NR1-NR1-NR2B-NR2B) or tri-heteromeric assemblies (such as NR1-NR1-NR2A-NR2B, NR1-NR1-NR2B-NR2D) (Cull-Candy and Leszkiewicz, 2004; Köhr, 2006). Additionally, massive excitatory and inhibitory neurons encode at least two types of NR2 subunits to give rise to di-heteromeric or tri-heteromeric NMDARs in the same neuron (Köhr, 2006). Speculatively, at least 80 kinds of NMDAR subtypes may exist in the central nervous system (Cull-Candy and Leszkiewicz, 2004).

The overstimulation of NMDARs generates massive Ca^2+^ influxes that overexcite neurons, finally leading to neuronal death (a pathological condition also known as excitotoxicity) (Lipton and Rosenberg, 1994; Lynch and Guttmann, 2002). The Aβ accumulation promotes the persistent Ca^2+^ influx through NMDARs, leading to neuronal excitotoxicity at the early stage AD (Figure 3) (Parameshwaran et al., 2008). Furthermore, the monomeric and oligomeric Aβ_42_ elevate the [Ca^2+^]_CYT_ level by activating the NR2B subunit of NMDARs in cultured cortical neurons (Ferreira et al., 2012). In turn, prolonged activation of extrasynaptic NMDARs, not synaptic NMDARs, promotes the production of Aβ in cultured cortical neurons (Lesné et al., 2005; Bordji et al., 2010). It reveals a positive feedback interaction between Aβ and NMDAR.

### NMDAR-Related Mitochondrial Ca^2+^ Uptake

Notably, compared with non-NMDARs or voltage-gated Ca^2+^ channels, NMDAR-related mitochondrial Ca^2+^ uptake is faster and tighter (Peng and Greenamyre, 1998). When neuronal [Ca^2+^]_CYT_ is elevated by NMDARs, the cytosol Ca^2+^ is segregated by the mitochondrial Ca^2+^ uptake; meanwhile, the mitochondrial Ca^2+^ transient persistently depolarizes the mitochondrial membrane potential (ΔΨ), causing the opening of the permeability transition pore (PTP) and further depolarising the ΔΨ, which parallels with the level of neuronal death (Schinder et al., 1996). Furthermore, under the circumstance in which the [Ca^2+^]_CYT_ elevated vastly, mitochondria divert their function from ATP synthesis to Ca^2+^ accumulation (Lipton and Rosenberg, 1994). Additionally, the lack of ATP synthesis affects Na^+^-K^+^-ATPase activity and results in plasm membrane depolarization, which alleviates the Mg^2+^ block of NMDARs and further activates NMDARs (Greene and Greenamyre, 1996). Mitochondrial Ca^2+^ uptake regulates NMDAR activity under a positive feedback mechanism.

Considering the fundamental role of NMDARs in normal synaptic functions, a complete antagonism of NMDARs generates the majority of side effects, such as severe memory impairment (Hardingham and Bading, 2010; Mota et al., 2014). Coincidentally, extrasynaptic NMDARs have been largely associated with neuronal excitotoxicity (Hardingham and Bading, 2010), and extrasynaptic NMDARs mainly contain NR2B subunits (Petralia, 2012). Thus, the selective blockage of extrasynaptic NR2B subunits may be a potential strategy to prevent synaptic dysfunction in AD (Mota et al., 2014).

## Presenilins are Related to Calcium Dyshomeostasis in Alzheimer's Disease

PSENs regulate Ca^2+^ signaling, and FAD-causing mutant PSENs perturb Ca^2+^ homeostasis (Leissring et al., 2000; LaFerla, 2002). Spatially, both PSEN1 and PSEN2 are mainly found on the ER membrane (Kovacs et al., 1996) and are widely expressed throughout the central nervous system (Cribbs et al., 1996). A series of FAD-causing mutant PSENs disrupt Ca^2+^ signaling (LaFerla, 2002). PSEN1-deficient neurons also reveal an increased [Ca^2+^]_CYT_ level after exposure to H_2_O_2_ (Nakajima et al., 2001). Indeed, PSENs do not contain any Ca^2+^-binding motif, so presenilins may interact with several Ca^2+^-binding proteins to regulate Ca^2+^ signaling (LaFerla, 2002).

### Cleaved Presenilins on the Plasma Membrane Possess γ-secretase Activity

The well-known function of PSENs is to provide the catalytic component of the γ-secretase complex, a membrane-embedded protease for several integral membrane proteins (De Strooper et al., 1998; Wolfe et al., 1999). PSEN has nine transmembrane domains (TMD) (Laudon et al., 2005). During maturation, PSEN is cleaved into a 30 KDa amino-terminal fragment (NTF) and a 20 KDa carboxy-terminal fragment (CTF) within a cytosol sizeable hydrophilic loop between TMD-6 and TMD-7 by endoproteolysis (Wolfe et al., 1999). Immature (or un-cleaved) presenilin holoproteins are localized on the ER membrane (Annaert et al., 1999). The endoproteolytic cleavage of PSEN holoproteins occurs on the ER membrane (Tandon and Fraser, 2002; Honarnejad and Herms, 2012). The cleaved PSEN (a heterodimer of NTF and CTF), together with anterior pharynx-defective 1 (APH-1), presenilin enhancer 2 (PEN-2), and nicastrin (all are ER transmembrane proteins), form the γ-secretase complex (De Strooper, 2003; Cheung et al., 2010; Honarnejad and Herms, 2012). The γ-secretase complex forms on the ER membrane and subsequently traffics to the Golgi apparatus, finally housed on the plasma membrane to generate Aβ peptide from APP (De Strooper et al., 2012; Honarnejad and Herms, 2012).

### Presenilin Holoproteins on the ER Membrane Function as Ca^2+^-Leaking Channels

Under the two suppositions that the sarcoplasmic/endoplasmic reticulum Ca^2+^ ATPase (SERCA) acts with 100% efficiency and the [Ca^2+^]_CYT_ level is 0.1 μM, the calculated upper limit value of the [Ca^2+^]_ER_ is 2,400 μM (Tu et al., 2006). In contrast, by directing measurement, the estimated [Ca^2+^]_ER_ level range is from 100 to 500 μM (Hofer, 1999). The leakiness of Ca^2+^ from the ER lumen to cytosol may explain the [Ca^2+^]_ER_ level difference mentioned above (Tu et al., 2006).

Tu and colleagues initially proposed the “presenilin calcium leak channel hypothesis”, in which the un-cleaved PSEN holoprotein functions as an ER passive Ca^2+^ leak channel independently from its γ-secretase activity, based on their sophisticated experiments with PSEN1/PSEN2 double knockout mouse embryonic fibroblasts (DKO-MEFs) (Figure 3) (Tu et al., 2006). The perturbed intracellular Ca^2+^ signaling in DKO-MEFs manifests as the potentiated amplitude of bradykinin-induced Ca^2+^ response, the exaggerated content of ionomycin-sensitive Ca^2+^ pool, and the reduced rate of thapsigargin-induced Ca^2+^ leak, compared with the wild-type control (Tu et al., 2006). Subsequently, in their rescue experiments, the expression of PSEN1_WT_ and PSEN2_WT_ successfully rescue Ca^2+^ signaling abnormalities, but PSEN1_M146V_ and PSEN2_N141I_ do not (Tu et al., 2006). Similarly, in planar lipid bilayers (BLM), the PSEN1_WT_ and PSEN2_WT_ can form a low-conductance divalent-cation-permeable channel, but PSEN1_M146V_ and PSEN2_N141I_ can not (Tu et al., 2006).

Quantitatively, the directly-measured [Ca^2+^]_ER_ level in DKO-MEFs (190 μM) is approximately 2-fold higher than it is in wild-type control (87 μM); moreover, it is calculated that PSENs account for 80% of the ER endogenous Ca^2+^-leaking ability (Tu et al., 2006). Additionally, PSEN1_D257A_, a mutation of catalytic aspartate indispensable for γ-secretase activity, forms a channel in BLM and alleviates all Ca^2+^ signaling perturbation in DKO-MEFs; specifically, PSEN1_ΔE9_ is a gain-of-function mutation that leads to Ca^2+^ over-leak from ER (Tu et al., 2006), likely contributing to elevated [Ca^2+^]_CYT_ and depleted [Ca^2+^]_ER_ (Bezprozvanny and Mattson, 2008). The presenilin calcium leak channel hypothesis is supported by Bandara and colleagues who investigated the role of PSEN2 in regulating [Ca^2+^]_ER_ using a fluorescence resonance energy transfer (FRET) probe (Bandara et al., 2013). The knockdown of PSEN2 significantly increases the [Ca^2+^]_ER_ level, and the overexpression of PSEN2 decreases the [Ca^2+^]_ER_ level (Bandara et al., 2013).

Adversely, Kasri and colleagues showed opposite conclusions: the increased Ca^2+^ leak from ER and the decreased [Ca^2+^]_ER_ level in the same DKO-MEFs model (Kasri et al., 2006). The presenilin calcium leak channel hypothesis is under suspicion by directly measuring ER Ca^2+^ dynamics (Shilling et al., 2012).

### FAD-Causing Mutant Presenilins Increase the Probability of InsP_3_R Opening

In 1994, Ito and colleagues first demonstrated that the InsP_3_-mediated Ca^2+^ liberation was potentiated in the skin fibroblast from AD patients (later known to harbor the PSEN1_A246Q_ mutation, a FAD-causing mutation) (Ito et al., 1994; LaFerla, 2002). In 1999, Leissring and colleagues found that the InsP_3_-mediated Ca^2+^ liberation was enhanced in the *Xenopus oocytes* model, expressing PSEN1_M146V_, PSEN2_N141I_, and PSEN2_M239V,_ all of which are FAD-causing mutations (Leissring et al., 1999a; Leissring et al., 1999b). The underlying mechanism is that FAD-causing mutant PSENs (PSEN1_M146L_, PSEN1_L166P_, PSEN1_A246E_, PSEN1_G384A_, PSEN2_N141I_) significantly elevate the probability of InsP_3_R opening compared with wild-type control (Cheung et al., 2008; Cheung et al., 2010). Interestingly, γ-secretase-eliminated mutant PSENs (PSEN1_D257A_, PSEN1_D385A_) also considerably enhance the InsP_3_R opening, which indicates that the γ-secretase activity of PSEN is not required for its influence on InsP_3_R opening (Cheung et al., 2010). Suppression of InsP_3_R1 expression genetically by 50% can normalize the enhanced InsP_3_R-mediated Ca^2+^ signaling associated with FAD-causing mutant PSENs (PSEN1_M146V_) and profoundly decreases both Aβ accumulation and tau protein hyperphosphorylation in cortical and hippocampal neurons of transgenic mice (Shilling et al., 2014). These lines of evidence support that the enhanced intraneuronal Ca^2+^ signaling by FAD-causing mutant PSENs is InsP_3_R dependent, and targeting the InsP_3_ signaling pathway could be a potential therapeutic strategy for FAD (Figure 3) (Shilling et al., 2014).

### Cytosolic Amino-Terminal Fragment of Presenilins Regulates RyR-Mediated Ca^2+^ Release

Payne and colleagues identified a novel mechanism under which the interaction between the cytosolic amino-terminal fragment of presenilin (PSEN-NTF_CYT_) and RyR regulates the Ca^2+^ signal from ER (Figure 3) (Payne et al., 2013). Physiological normal Ca^2+^ concentration (10 nM < [Ca^2+^]_CYT_ < 1 μM) and pathological high Ca^2+^ concentration ([Ca^2+^]_CYT_ > 10 μM) are required for the cytosolic amino-terminal fragment residues 1–82 of presenilin-1 (PSEN1-NTF_CYT1-82_) and the cytosolic amino-terminal fragment residues 1–87 of presenilin-2 (PSEN2-NTF_CYT1-87_) to bind RyR, respectively (Hayrapetyan et al., 2008; Rybalchenko et al., 2008; Payne et al., 2013). After PSEN1-NTF_CYT1-82_ binding RyR at normal [Ca^2+^]_CYT_, the single RyR opening probability and mean currents are potentiated, causing an increased rate of Ca^2+^ release (Figure 3) (Rybalchenko et al., 2008; Payne et al., 2013). Hence, the whole-neuron net Ca^2+^ release from ER is reduced due to the inhibitory Ca^2+^ concentration being reached in a shorter time (Rybalchenko et al., 2008; Payne et al., 2013). After PSEN2-NTF_CYT1-87_ binding RyR at high [Ca^2+^]_CYT_, the low-affinity inhibitory Ca^2+^-binding site is blocked, resulting in more elevated [Ca^2+^]_CYT_ is required to close the RyR, which represent a potential feedforward mechanism of Ca^2+^ dysregulation (Hayrapetyan et al., 2008; Payne et al., 2013).

## Discussion

For receiving information about the changing environment, cells evolved the ability to signal (Clapham, 2007). Even though the precise definition of the signal is still controversial, it is recently stated that anything that changes could be a signal (Chakravorty, 2018). Ca^2+^ is elegantly manipulated by cells, particularly neurons, as a second messenger (Clapham, 2007). The unequal distribution of ions inside and outside neurons, such as K^+^, Na^+^, and Cl^−^, keeps the cellular function by generating the resting membrane potential and holds the neuronal volume by maintaining the osmotic balance (Byrne et al., 2014). It is widely known that the large gradient between extracellular and intracellular Ca^2+^ concentration levels is the most significant among particles with electrical charges. Cells possess numerous molecular machinery to regulate the Ca^2+^ distribution spatially and temporally; simultaneously, numbers of biochemical reactions are controlled by intracellular Ca^2+^. Therefore, the Ca^2+^ signal can transmit various information throughout the cells, and neurons are no exception (Berridge et al., 2000).

The generation and termination of the Ca^2+^ signal are featured a**s** increasing [Ca^2+^]_CYT_ and decreasing [Ca^2+^]_CYT_, respectively (Miller, 1991). Multiple Ca^2+^ channels exist in the various compartment of neurons to perform separate functions (Berridge et al., 2000). The [Ca^2+^]_CYT_ is changed by extracellular stimuli through directly activating the gated Ca^2+^ channels on the plasma membrane or indirectly triggering the Ca^2+^-release channels on intracellular Ca^2+^ stores (Takei et al., 1992). In turn, Ca^2+^, released from ER, can alter transmembrane potential and regulate the excitability of neurons (Berridge, 1998). Spatiotemporally different Ca^2+^ signals modulate a series of neuronal functions, such as neurotransmitter release, post-tetanic potentiation, long-term potentiation (LTP), and long-term depression (LTD) (Purves et al., 2018). For example, large and fast Ca^2+^ signals evoke LTP, and small and slow Ca^2+^ signals trigger LTD (Purves et al., 2018). For neurons under physiological conditions, [Ca^2+^]_CYT_, [Ca^2+^]_ER_, and [Ca^2+^]_MT_ are at a subtle equilibrium level. Both ER and mitochondria can shape the [Ca^2+^]_CYT_. In addition, the Ca^2+^ in the ER lumen can transmit into the mitochondrial matrix through ERMCS (Wu et al., 2018). Collectively, maintaining the Ca^2+^ homeostasis is vital for neurons.

Dysregulation in Ca^2+^ signaling has been reported in neurodegenerative diseases, such as AD, Parkinson’s disease (PD), and Huntington’s disease (HD) (Bezprozvanny and Mattson, 2008; Sheng and Cai, 2012; Pchitskaya et al., 2018). The [Ca^2+^]_ER_ is overfilled in AD, whereas depleted in PD and HD (Pchitskaya et al., 2018). In *Caenorhabditis elegans*, mutations in the SEL-12 (the PSEN ortholog) can elevate the [Ca^2+^]_MT_ level, and reducing the Ca^2+^ signal from ER to mitochondria normalizes the [Ca^2+^]_MT_ level and the mitochondrial function (Sarasija et al., 2018). In neurons, mitochondria dysfunction is recognized as a final pathway in neurodegeneration (Friedman et al., 2010; Rizzuto et al., 2012). Area-Gomez and colleagues observed that PSENs are abundant in ERMCS (Area-Gomez et al., 2009), later the same research team demonstrated that mutations in PSEN1, PSEN2, and APP can upregulate the function of ERMCS (Area-Gomez et al., 2012). Moreover, variations in ERMCS likely influence the cellular Ca^2+^ homeostasis (Area-Gomez et al., 2012).

The present review summarizes the intracellular Ca^2+^ signaling regulated by molecular machinery on cellular membrane systems and the Ca^2+^ dyshomeostasis linked to Aβ and presenilins. Connecting the amyloid hypothesis with the calcium hypothesis may further the understanding of Alzheimer’s disease pathogenesis. At ER and mitochondria levels, understanding the regulation of cellular Ca^2+^ signaling and the mechanism underlying neuronal Ca^2+^ dyshomeostasis in AD may provide therapeutic targets for chronic neuronal degeneration disease in the central nervous system.
